# MAKING OLDER ADULT FALL PREVENTION A ROUTINE PART OF HEALTHCARE IN TRIBAL CLINICAL SETTINGS

**DOI:** 10.1093/geroni/igad104.3703

**Published:** 2023-12-21

**Authors:** Gwen Bergen, Leo Egashira, Myra Parker, Shakiera Causey, Yara Haddad

**Affiliations:** Centers for Disease Control and Prevention, Atlanta, Georgia, United States; Seven Directions Public Health Institute, Seattle, Washington, United States; Seven Directions Public Health Institute, Seattle, Washington, United States; National Network of Public Health Institutes, New Orleans, Louisiana, United States; Centers for Disease Control and Prevention, Atlanta, Georgia, United States

## Abstract

Over one-third of American Indian and Alaska Native (AI/AN) elders (age 65+) report falling annually. This percentage is higher compared to other race/ethnic groups except for White non-Hispanic older adults. CDC’s Stopping Elderly Accidents, Deaths, and Injuries (STEADI) initiative provides guidance for making elder fall prevention a routine part of clinical care. The presentation will describe a survey of healthcare providers serving AI/AN populations to gauge their readiness to adopt STEADI. Additionally, it will focus on a culturally informed approach to implementing STEADI within tribal settings. The National Network of Public Health Institutes and Seven Directions, A Center for Indigenous Public Health, surveyed a convenient sample of healthcare providers in clinics serving AI/AN patients to assess providers’ knowledge and acceptability of fall prevention best practices, efforts to prevent falls, and receptiveness to receiving fall prevention training and support. The final sample included 94 healthcare providers from 27 clinics. Less than half of those surveyed reported screening elders for fall risk. Only 22% were familiar with STEADI. Most (84%) providers were willing to take part in fall prevention with additional training. Based on these findings CDC is funding two tribes to use an indigenous evaluation approach to improve their tribal health center’s elder fall prevention capacity through evidence-based clinical and clinical-community linked strategies. Results from these activities will be used to outline a best practices guide focused on coordinated care for fall prevention and tailored to the varied environments, governments, cultures, and capacity for public health action in tribal health centers.

